# Editorial

**Published:** 1844-10-05

**Authors:** 


### THE MEDICAL EXAMINER.

PHILADELPHIA, OCT. 5, 1844.

### NEW ORLEANS MEDICAL JOURNAL.

What has become of this Journal? We received the first number, (May, 1844,) but have had none since, nor can we learn that it has been received by any one in this city. Will our brethren of N. O. look to it?

---

### QUACKERY AT A DISCOUNT.

Among the advertisements on the cover of a late number of the *London Lancet*, the following is conspicuous: "Hydropathy.—As many members of the Medical profession have now satisfied themselves of the value of Hydropathy, it is no longer expedient that a private individual should seek to vindicate the cause. Mr. Richard Beamish is, therefore, willing to resign his position at Prestburg, with his establishment at Field House, and all Hydropathic Appliances, upon any moderate terms."

The meaning of this we take to be, that, so "many members of the Medical Profession," having repudiated a profession which had already repudiated them, and betaken themselves to the rather fashionable humbug in which he had engaged, he finds it no longer profitable, and is therefore willing to "resign his position," with all its "hydropathic appliances, upon any moderate terms"—or, as the shop-keepers advertise upon their windows: "*Selling off below cost, to close business.*"

It will not be long till "Mr. Richard Beamish" will have followers in this as in his former example. We have heard of some Homœopathists lately, who, like him, have been engaged in the very disinterested business of "vindicating the cause," until "many members of the medical profession have now satisfied themselves of its value," and the *philanthropists* are therefore now seeking Hydropathic establishments, in order next to vindicate that.

